# Supplementary material for: Sodium-Glucose Cotransporter-2 Inhibitors in Liver Cirrhosis: A Systematic Review of Their Role in Ascites Management, Slowing Disease Progression, and Safety
Source: Int J Mol Sci. 2025 May 16;26(10):4781. doi: 10.3390/ijms26104781 (PMC12112422; doi:10.3390/ijms26104781)
Supplement: Supplementary file 1 [file ijms-26-04781-s001.zip › ijms-3573921-supplementary.pdf]

Table S1. Search Results by Database And/Or Registrar.

Database	Results	Platform
Embase	196	Embase.com (Elsevier)
MEDLINE	61	PubMed (NCBI, National Libraries of Medicine)
Cochrane Central Register of Controlled Trials	42	Cochrane Library (Wiley)
Web of Science Core Collection	95	Web of Science (Clarivate)
Total	394	
with duplicates removed	293	

Table S2. Search Strategy: Search Strategy Constructed in Embase Translated to Other Databases

Keyword search terms:
gliflozin* OR gliflozin-derivative* OR SGLT2-inhibitor* OR sodium-dependent-glucose-cotransporter-2-inhibitor* OR sodium-glucose-co-transporter-2-inhibitor* OR sodium-glucose-transporter-2-inhibitors* OR sodium-glucose-cotransporter-2-inhibitor* OR SGLT-2-inhibit* OR SGLT2i OR SGLT2is OR SGLT2-I OR atigliflozin OR bexagliflozin OR canagliflozin OR dapagliflozin OR empagliflozin OR enavogliflozin OR ertugliflozin OR ipragliflozin OR licogliflozin OR luseogliflozin OR mizagliflozin OR remogliflozin OR sergliflozin OR sotagliflozin OR tofogliflozin
cirrhosis OR alcohol*-liver* OR decompensated-liver* OR hepatic-decompensat* OR liver-decompensat*
Embase.com Searches (with Emtree Search headings). Do not use subheadings unless you intend to exclude Conference Abstracts<br>‘sodium glucose cotransporter 2 inhibitor’/syn<br>‘liver cirrhosis’/syn NOT ((‘fatty liver’/exp/mj OR NAFLD:ti OR MAFLD:ti OR MASLD:ti OR fatty-liver:ti) NOT (‘liver cirrhosis’/exp/mj NOT cirrhos*:ti))<br>NOT ([animals]/lim NOT [humans]/lim)<br>NOT (‘conference review’/it OR ‘editorial’/it OR ‘letter’/it OR ‘note’/it OR ‘review’/it OR ‘short survey’/it OR ‘tombstone’/it OR ‘case report’/de OR ‘meta analysis’/de OR ‘meta analysis topic’/de OR ‘systematic review’/de OR ‘systematic review topic’/de)
MEDLINE in PubMed (with MeSH headings). Use OVID MEDLINE if you are using proximity operators.<br>“Liver Cirrhosis”[Mesh] NOT ((“Fatty Liver”[Majr] OR NAFLD[ti] OR MAFLD[ti] OR MASLD[ti] OR fatty-liver[ti]) NOT (“Liver Cirrhosis”[Majr] OR cirrhos*[ti]))<br>“Sodium-Glucose Transporter 2 Inhibitors”[Mesh] OR “Sodium-Glucose Transporter 2 Inhibitors” [Pharmacological Action]<br>NOT (“animals”[mesh] NOT “humans”[mesh])<br>NOT (“case reports”[Publication Type] OR “comment”[Publication Type] OR “editorial”[Publication Type] OR “guideline”[Publication Type] OR “introductory journal article”[Publication Type] OR “meta analysis”[Publication Type] OR “news”[Publication Type] OR “retracted publication”[Publication Type] OR “review”[Publication Type] OR “systematic review”[Publication Type])

Cochrane Central Register of Controlled Trials. Use MEDLINE search but “Term”[MeSH] > [mh “Term”]. Limit to “Trials”, so results are automatically limited to Controlled Trial studies and Trial Registries, so no need for publication type limits.
Keywords for Global Index Medicus—remove truncation from keywords. Use “” around phrases.

Table S3. PRISMA Checklist.

Section and Topic	Item #	Checklist item	Location where item is reported
**TITLE**			
Title	1	Identify the report as a systematic review.	1-4
**ABSTRACT**			
Abstract	2	See the PRISMA 2020 for Abstracts checklist.	13-30
**INTRODUCTION**			
Rationale	3	Describe the rationale for the review in the context of existing knowledge.	44-51, 93-95
Objectives	4	Provide an explicit statement of the objective(s) or question(s) the review addresses.	96-99
**METHODS**			
Eligibility criteria	5	Specify the inclusion and exclusion criteria for the review and how studies were grouped for the syntheses.	111-149
Information sources	6	Specify all databases, registers, websites, organisations, reference lists and other sources searched or consulted to identify studies. Specify the date when each source was last searched or consulted.	101-110
Search strategy	7	Present the full search strategies for all databases, registers and websites, including any filters and limits used.	S2; 598-599
Selection process	8	Specify the methods used to decide whether a study met the inclusion criteria of the review, including how many reviewers screened each record and each report retrieved, whether they worked independently, and if applicable, details of automation tools used in the process.	111-123
Data collection process	9	Specify the methods used to collect data from reports, including how many reviewers collected data from each report, whether they worked independently, any processes for obtaining or confirming data from study investigators, and if applicable, details of automation tools used in the process.	126-159
Data items	10a	List and define all outcomes for which data were sought. Specify whether all results that were compatible with each outcome domain in each study were sought (e.g., for all measures, time points, analyses), and if not, the methods used to decide which results to collect.	136-159
	10b	List and define all other variables for which data were sought (e.g., participant and intervention characteristics, funding sources). Describe any assumptions made about any missing or unclear information.	126-159
Study risk of bias	11	Specify the methods used to assess risk of bias in the included studies, including details of the tool(s) used, how many reviewers assessed each study and whether	150-159

Section and Topic	Item #	Checklist item	Location where item is reported
assessment		they worked independently, and if applicable, details of automation tools used in the process.	
Effect measures	12	Specify for each outcome the effect measure(s) (e.g., risk ratio, mean difference) used in the synthesis or presentation of results.	149-150
Synthesis methods	13a	Describe the processes used to decide which studies were eligible for each synthesis (e.g., tabulating the study intervention characteristics and comparing against the planned groups for each synthesis (item #5)).	126-134
	13b	Describe any methods required to prepare the data for presentation or synthesis, such as handling of missing summary statistics, or data conversions.	N/A
	13c	Describe any methods used to tabulate or visually display results of individual studies and syntheses.	149-150
	13d	Describe any methods used to synthesize results and provide a rationale for the choice(s). If meta-analysis was performed, describe the model(s), method(s) to identify the presence and extent of statistical heterogeneity, and software package(s) used.	157-160
	13e	Describe any methods used to explore possible causes of heterogeneity among study results (e.g., subgroup analysis, meta-regression).	131-134
	13f	Describe any sensitivity analyses conducted to assess robustness of the synthesized results.	N/A
Reporting bias assessment	14	Describe any methods used to assess risk of bias due to missing results in a synthesis (arising from reporting biases).	151-155
Certainty assessment	15	Describe any methods used to assess certainty (or confidence) in the body of evidence for an outcome.	160-162
**RESULTS**			
Study selection	16a	Describe the results of the search and selection process, from the number of records identified in the search to the number of studies included in the review, ideally using a flow diagram.	163-170; Figure 1
	16b	Cite studies that might appear to meet the inclusion criteria, but which were excluded, and explain why they were excluded.	166-170
Study characteristics	17	Cite each included study and present its characteristics.	165-166; Table 1 & 2
Risk of bias in studies	18	Present assessments of risk of bias for each included study.	263-288; Table 3
Results of individual studies	19	For all outcomes, present, for each study: (a) summary statistics for each group (where appropriate) and (b) an effect estimate and its precision (e.g., confidence/credible interval), ideally using structured tables or plots.	289-297; Table 4 Figure 2
Results of syntheses	20a	For each synthesis, briefly summarise the characteristics and risk of bias among contributing studies.	Table 3 331-546
	20b	Present results of all statistical syntheses conducted. If meta-analysis was done, present for each the summary estimate and its precision (e.g., confidence/credible interval) and measures of statistical heterogeneity. If comparing groups, describe the direction of the effect.	N/A
	20c	Present results of all investigations of possible causes of heterogeneity among	N/A

Section and Topic	Item #	Checklist item	Location where item is reported
		study results.	
	20d	Present results of all sensitivity analyses conducted to assess the robustness of the synthesized results.	N/A
Reporting biases	21	Present assessments of risk of bias due to missing results (arising from reporting biases) for each synthesis assessed.	N/A
Certainty of evidence	22	Present assessments of certainty (or confidence) in the body of evidence for each outcome assessed.	Figure 2
**DISCUSSION**			
Discussion	23a	Provide a general interpretation of the results in the context of other evidence.	311-330
	23b	Discuss any limitations of the evidence included in the review.	570-583
	23c	Discuss any limitations of the review processes used.	570-572
	23d	Discuss implications of the results for practice, policy, and future research.	547-569; 581-592
